# Change in multidimensional problems and quality of life over three months after HIV diagnosis: a multicentre longitudinal study in Kenya and Uganda

**DOI:** 10.1186/s12879-019-3855-0

**Published:** 2019-03-12

**Authors:** Victoria Simms, Julia Downing, Eve Namisango, R. Anthony Powell, Faith Mwangi-Powell, Irene J. Higginson, Richard Harding

**Affiliations:** 1 London School of Hygiene and Tropical Medicine, Keppel Street, London, WC1E 7HT England; 2 King’s College London, Florence Nightingale Faculty of Nursing Midwifery and Palliative Care, Cicely Saunders Institute, Department of Palliative Care, Policy and Rehabilitation, Bessemer Road, London, SE5 9PJ England; 3 African Palliative Care Association, P. O. Box 72518, Plot 95, Dr Gibbons Road, Makindye Kampala, Uganda; 4 MWAPO Health Development Group, Nairobi, Kenya

**Keywords:** HIV, Newly diagnosed, Patient-report, Adjustment

## Abstract

**Background:**

Evidence on patient-reported outcomes of newly diagnosed HIV patients is scarce, and largely cross-sectional. This prospective cohort study describes the prevalence of, and changes in, patient-reported outcomes in the three months after HIV diagnosis, in 11 HIV outpatient centres in Kenya and Uganda.

**Methods:**

Adults were recruited within 14 days of result, completing self-report measures four times at monthly intervals. Multilevel mixed-effects linear regression (quality of life continuous outcomes) and ordinal logistic regression (symptoms and concerns categorical outcomes) modelled change over time, with repeated observations grouped within individuals adjusted for demographic/clinical characteristics, and multiple imputation for missing data.

**Results:**

438 adults were enrolled and 234 (53·4%) initiated ART. Improvement was found for MOS-HIV physical health (from 46·3 [95% CI 45·1–47·3], to 53·7 [95% CI 52.8–54·6], *p* < 0.001), and mental health (from 46·4 [95% CI 45·5–47·3] to 54·5 [95% CI 53·7–55·4], p < 0.001). POS subscale ‘interpersonal problems’ improved but remained burdensome (OR = 0·91, 95% CI = 0·87–0·94, p < 0.001; 22·7% reported severe problems at final time point). The scores for the existential POS subscale (OR = 0·95, 95% CI = 0.90–1·00, *p* = 0.056) and physical/psychological problems POS subscale (OR = 0·97, 95% CI = 0.92–1·02, *p* = 0.259) did not improve.

Participants who initiated ART had worsening physical/psychological (OR = 0·64, 95% CI = 0·41–0·99, p = 0·045) and interpersonal problems (OR = 0·64, 95% CI = 0·42–0·96, p = 0·033).

**Conclusion:**

Although some self-reported outcomes improve over time, burden of interpersonal problems remains substantial and existential concerns do not improve.

## Background

Increasing numbers of people will test HIV positive and enter care due to efforts made to meet the UNAIDS 90–90-90 targets of 90% of people with HIV in care, 90% of those in care on antiretroviral therapy, and 90% of treated patients virally suppressed [1]. In resource-constrained settings where the HIV disease burden is greatest, health systems and providers are under pressure, and a paradigm shift is needed to enable an adequate, sustainable response [2]. In order to achieve viral suppression, patients are required to adhere to ART and remain engaged with treatment and care facilities. The current model of HIV care is delivered in overstretched systems with weak infrastructure and high patient numbers, and these challenges have been associated with attrition from ART programmes [3].

People recently diagnosed with HIV report physical [4], psychological [5] and social support problems [6]. These problems have been identified by HIV outpatients in East Africa as contributors to poor attendance and treatment adherence [7]. In a South African study of people recently diagnosed with HIV, 14·8% met the criteria for post-traumatic stress disorder, which is associated with worse functional health status [8]. This may limit patients’ ability to absorb information and make critical decisions about their care [9]. Conversely, the phenomenon of post-traumatic growth has also been described among people diagnosed with serious health conditions, and the individual may report improved relationships, enhanced appreciation for life, and a greater sense of personal strength [10].

A systematic review concluded that the lack of longitudinal studies has prevented understanding of the individual response to a HIV diagnosis over time, in terms of quality of life, physical and psychosocial outcomes [11]. A number of gaps were identified in the evidence, principally: lack of measures of emotional support needs, omission of spiritual distress, lack of studies that recruited outpatients in low-income countries, use of staff proxy rather than self-report, lack of measurement of problems across domains, small sample sizes, lack of repeated measures to appraise adjustment to diagnosis, and no longitudinal analysis methods.

Our cross-sectional analysis of 438 adults newly diagnosed with HIV (i.e. within 14 days) in 11 outpatient centres in Kenya and Uganda reported the following main problems: help and advice (64·7% scoring at the worst end of the scale, i.e., 4–5 on a scale of 0–5); sharing feelings (53·0%); spiritual distress (19·2%); feeling life is not worthwhile (10·0%) [12]. For these problems and additional measures of health-related quality of life, all outcomes were independent of CD4 count or ART eligibility. To retain these individuals in care, we must understand how their problems change immediately following diagnosis so that responsive care can be planned.

The aim of this paper is to measure change in patient-reported outcomes in the first three months following HIV diagnosis. The study objectives were to: i) determine eligibility for ART at diagnosis and uptake in the 3 months following diagnosis; ii) measure change in patient-reported outcomes, iii) identify clinical and demographic predictors of change.

## Methods

### Design

A secondary analysis was carried out on longitudinal data from an observational cohort study conducted by the authors as part of a mixed-methods evaluation of care and support for the President’s Emergency Plan for AIDS Relief (PEPFAR). The study protocol has previously been published [13]. The full cohort consisted of approximately 120 adult HIV-positive outpatients from each of 12 rural and urban health facilities in Kenya and Uganda, enrolled in 2008 by consecutive sampling and interviewed four times at monthly intervals [14]. The inclusion criteria were age 18 or over; aware of their HIV diagnosis; and being new to care or presenting with a new problem/symptom [13, 15]. Of these 1337 participants, those enrolled within 14 days following their HIV diagnosis were selected for this secondary analysis.

### Procedure

438 participants were enrolled within 14 days following their HIV diagnosis at one of 11 clinical settings in Kenya and Uganda (the twelfth had no newly diagnosed patients), and were included in this analysis. Participants were either diagnosed at the health facility where the study was based, or diagnosed at a smaller health post/mobile testing centre and referred to the recruiting facility for their HIV care.

Self-report data using validated patient-centred outcome measures were collected at baseline and for 3 subsequent monthly time points. The MOS-HIV (Medical Outcomes Scale for HIV) is a widely used 35-item health-related quality of life questionnaire [16] validated in many countries including Uganda [17, 18]. Multidimensional problems (physical, psychological, social and spiritual) were measured using the APCA African Palliative Outcomes Scale (POS), designed and validated in sub-Saharan Africa [19]. The POS is a 10-item Likert questionnaire about multidimensional problems in the previous 3 days, with each item scored 0–5. A demographic questionnaire was completed at baseline only, and the date and results of a CD4 test (up to six months after HIV diagnosis) and World Health Organization (WHO) HIV stage were extracted from patients’ records. Baseline data collection also included the ECOG, a widely used single item measure of physical function [20]. Questionnaires were translated from English into Swahili, Luo, Luganda and Runyakitara, then piloted.

### Analysis

Analysis was conducted using Stata v14.0. MOS-HIV item scores were converted into continuous 0–100 summary scores for mental and physical health, with higher scores indicating better health [17, 21]. The POS was analysed using three previously validated factors: physical/psychological well-being, interpersonal well-being, and existential well-being [22, 23]. Poverty quintile was calculated in line with the DHS method using variables including house construction, possession of items, and fuel supply [24]. APCA African POS factors were categorised into mild/none (score 0–4), moderate problems [5–7] and severe problems (≥8). The time variable was defined as number of weeks since enrolment in care. Interviews were scheduled for one month apart but participants frequently attended at longer intervals, so actual time was used rather than number of visits. Visits more than 20 weeks after enrolment were excluded as outliers. Multilevel mixed-effects linear regression with robust standard errors was used to fit models for the subscale continuous variables physical health score and mental health score, with observations grouped within individuals. For the ordinal POS factor variables, multilevel mixed-effects ordinal logistic regression was used. Co-variates were selected a priori and were either binary (gender, ART initiation, country) or ordinal, fitted as continuous (age category, physical function, poverty quintile). ART initiation was defined as having initiated ART during follow-up. To impute missing data we used multiple imputation with 10 iterations on the same covariates plus baseline score, having set the data as cross-sectional time series with individual ID to identify the person and time since enrolment for the time.

## Results

### Sample characteristics

Baseline characteristics of the sample have been previously described in detail [12]. Of the 438 newly diagnosed participants, 270 (61·6%) were women, the age range was 18–59 (mean 32·9); just over half (56·4%, *n* = 247) were enrolled in Kenya. Age distribution was 20·3% aged 18–25, 63·0% aged 26–40 and 16·7% aged 41–59. The majority (69·9%, *n* = 306) were physically fully active (ECOG score 0), with 23·3% (*n* = 102) scoring 1 and 6·8% (n = 30) scoring more than 1. WHO stage was obtained for 198 participants, with 26% Stage I, 36% Stage II, 31% Stage III and 7% Stage IV.

**Fig. 1:**
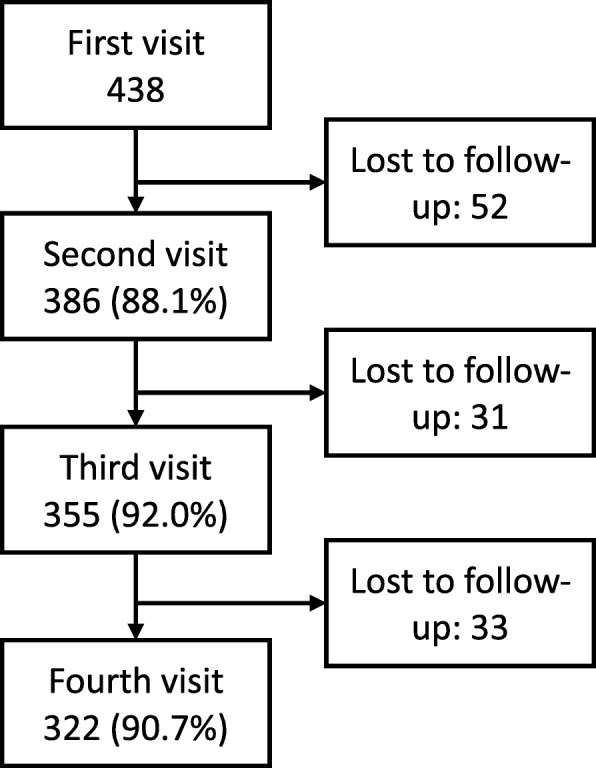
Number of participants per visit

Within 20 weeks, out of 438 participants, 52 (11·9%) completed only one interview, 31 (7·1%) completed two, 33 (7·5%) completed three and 322 (73·5%) completed all four (Fig. 1), making 1501 research data collection visits out of a possible 1752 (85·7%). Out of 1501 visits, 438 were on the day of enrolment, by definition. 332 (22·1%) were 1–40 days after enrolment, 405 (27·0%) 41–80 days after enrolment, 312 (20·8%) 81–120 days after enrolment and 14 (0·9%) 121–242 days after enrolment.

### Attendance and treatment

ART eligibility was not directly recorded at data collection so it was inferred later based on CD4 count and WHO stage where these were available. In line with the national guidelines in use at the time of data collection, patients with a CD4 count below 350 cells/μl or a WHO stage 4 were eligible for ART [25]. Patients with both CD4 count ≥350 cells/μl and WHO stage 1–3 were not eligible. Using these criteria, ART eligibility could be established for 263/438 people (Table 1). 207 (78.7%) were eligible for ART at enrolment (CD4 < 350 or WHO stage 4). Out of 207 participants eligible for ART, 154 (74.4%) initiated ART within the study period and 53 (25·6%) did not. Of the 53 who did not initiate,16 only attended one appointment at the facility and another 8 dropped out before completing all 4 appointments. For 5/53 who did not initiate ART, their CD4 test was delayed, so their eligibility was not confirmed during follow-up. 21 people took ART at least once despite not being eligible according to CD4 count and WHO stage. Out of 175 participants whose ART eligibility was unknown according to available data on file, 59 initiated ART.

**Table 1:** ART eligibility and initiation

	Initiated ART n	Never initiated n	Total n
Eligible	154	53	207 (47·3%)
Not eligible	21	35	56 (12·8%)
Eligibility unknown	59	116	175 (40·0%)
Total	234 (53·4%)	204 (46·6%)	438 (100%)

### Change in patient-reported outcomes

Participants with fewer than 3 follow-up observations had mean baseline physical and mental health scores 3 points lower than those of participants who had no missing data, and were also more likely to report Existential problems.

At baseline, mean score on the physical health score of the MOS-HIV was 46·3 (standard deviation 12.0). Mental health score at baseline was 46·4 (standard deviation 9·9). A multi-level model with no covariates except for time showed that physical health score improved by 0.5 per week (95% CI 0·4–0·6) and mental health score by 0·6 per week (95% CI 0·5–0·6).

**Fig. 2:**
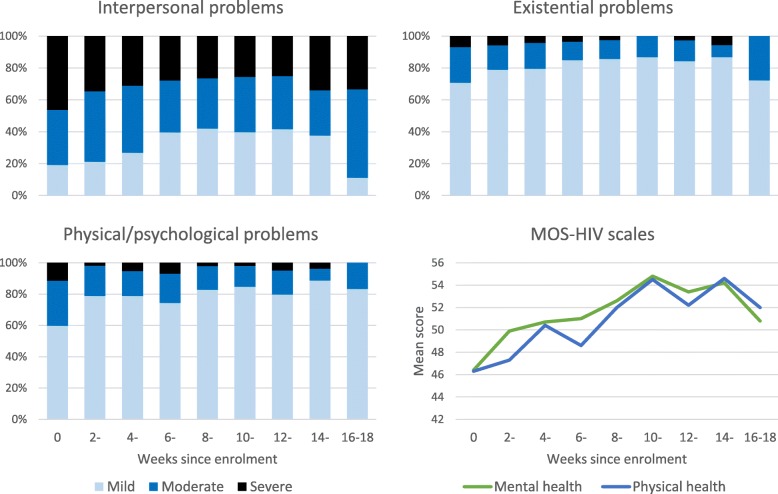
Multidimensional problems and health-related quality of life over time

The prevalence of severe and moderate problems as measured by the POS decreased over time for all subscales (Fig. 2). However, interpersonal problems were the most pervasive. Even after four clinic visits 74/326 (22·7%) participants still reported severe interpersonal problems and less than half (43·6%) reported mild/no problems.

After controlling for sociodemographic and health factors, on the MOS-HIV subscales, mental and physical health scores both improved over time after recruitment into care. On POS, Interpersonal problems subscale improved over time. Existential problems improved, but with only borderline significance (*p* = 0.056). No statistically significant improvement was found for the physical/psychological subscale.

### Predictors of change on patient-reported outcomes

Limited physical function at baseline was associated with worsening physical health score but improvement in the interpersonal subscale (Table 2). Older age was associated with improvement in mental health score and existential problems over time. Gender was not associated with any outcome.

**Table 2:** Mixed-effects models for multidimensional problems and health-related quality of life over time

		physical health score		mental health score		APCA POS Physical/ psych. Problems		APCA POS Interpersonal problems		APCA POS Existential problems	
Variable	Reference group	Coeff (95% CI)	p	Coeff (95% CI)	p	OR (95% CI)	p	OR 95% CI)	p	OR (95% CI)	p
Week	0	0·28 (0·16, 0·40)	< 0·001	0·35 (0·25, 0·45)	< 0·001	0·97 (0·92, 1·02)	0·259	0·91 (0·87, 0.94)	< 0·001	0·95 (0·90, 1·00)	0·056
Physical function	Fully active	−8·69 (−10·18, −7·21)	< 0·001	−4·93 (−6·09, −3·76)	< 0·001	4·29 (2·94, 6·26)	< 0·001	0·86 (0·63, 1·19)	0·373	2·92 (1·89, 4.50)	< 0·001
Gender	Male	0·81 (−0·48, 2·09)	0·218	0·23 (−1·11, 1·58)	0·733	1·22 (0·75, 2·00)	0·420	0·92 (0·59, 1·44)	0·717	0·69 (0·39, 1·23)	0·208
Age group	18–25	0·50 (−0·58, 1·59)	0·365	1·17 (0·03, 2·30)	0·045	1·28 (0·87, 1·87)	0·213	0·76 (0·54, 1·09)	0·133	0·65 (0·41, 1·03)	0·067
Poverty quintile	Poorest	0·46 (0·04, 0·88)	0·033	0·38 (−0.05, 0·81)	0·082	0·76 (0·64, 0·91)	0·002	1·19 (1·02, 1·39)	0·031	0·88 (0·71, 1·07)	0·204
ART	No ART	0·71 (−0·51, 1·92)	0·256	1.44 (0.11, 2.76)	0·033	0·51 (0·31, 0·82)	0·005	0·61 (0·39, 0·95)	0·028	0·86 (0·49, 1·52)	0·605
Country	Kenya	−0·98 (−2·19, 0·24)	0·114	−2·48 (−3·81, −1·15)	< 0·001	1·70 (1·07, 2·71)	0·026	1·01 (0·66, 1·55)	0·969	3·05 (1·72, 5·42)	< 0·001

Compared to the economically poorest, those with greater wealth were likely to have improved physical and mental health score and fewer physical/psychological or existential problems over time. Compared to Kenya, participants in Uganda had less improvement in physical or mental health score over time, and more physical/psychological and existential problems.

With respect to ART, initiation during the study period was not associated with improvement in physical or mental health scores or existential problems, but was associated with improvement in both physical/psychological problems and interpersonal problems.

## Discussion

This novel longitudinal multi-centred cohort study found that, for adults newly diagnosed with HIV infection in East Africa, statistically significant improvement was found for both physical and mental health-related quality of life (when controlling for patient characteristics). While there was also improvement in interpersonal and existential problems, no statistically significant improvement was found for physical/psychological problems.

This study has advanced the evidence by addressing the conclusions of a systematic review which revealed an absence of data to determine how people newly diagnosed with HIV change in their self-report health status over time [11]. This is timely, given the current policy to greatly increase the proportion of people infected with HIV who receive a test result, initiate therapy and remain in care to achieve viral suppression.

The association of POS scores and health-related quality of life outcomes with demographic variables at baseline have been presented previously in a cross-sectional study [12]. This study shows the association of variables with change over time, adjusting for baseline values. In the previous paper, the seven POS outcomes were analysed separately. Since then, factor analysis has determined the POS is composed of three factors [22], which have been used as outcomes here.

Our descriptive analysis showed that the prevalence of severe and moderate physical/psychological problems, as measured by the POS, decreased over time. This association, however, was no longer significant in the multivariate analysis after missing datapoints had been imputed through multiple imputation. There was a rise in prevalence of severe problems between the second and third follow-up visits, which may have weakened the association. Missingness was associated with lower baseline scores for physical and mental health. Without multiple imputation, most findings were similar but physical function was closely associated with all outcomes. The likely explanation is that participants with poor physical function were the most likely not to return.

Gender was not associated with any outcomes. In a longitudinal study of 1274 people with HIV in Uganda, women were at increased risk of poor mental health score (if on ART) and poor physical health score (if not initiated on ART) [26]. Depression was associated with worse physical health score over time, whether patients were on ART or not. There was no association between change in CD4 count over time and physical or mental health score [27], possibly because CD4 count was relatively high at baseline (> 350 for 80% of participants), or possibly because health scores reflect a more complex state of quality of life that is not closely tied to immunological function.

The Kenya population had a lower CD4 count distribution, although this is difficult to compare because almost half the Uganda sample did not have a CD4 count in the dataset. This challenge in lack of CD4 count on file has been previously reported [28], and is a concern for the current policy of increasing the proportion of eligible people on ART. The 11 sites varied in their settings, services and populations. Notably, two of the Uganda sites were TASO (The AIDS Support Organisation) centres, which may offer a different balance of services.

Interestingly, ART was not associated with improvement in physical health score over time, but there was an association with the physical/psychological problems and interpersonal problems subscales. This may reflect the relatively brief time period, and also any improvement in physical health due to viral suppression may have been offset by initial treatment-related side effects. It is important to note that while treatment did not in the short term improve existential problems, it was associated with improvement in interpersonal problems. This is likely due to the increased communication required to begin and maintain treatment.

Our study confirms earlier findings that health-related quality of life is worst at diagnosis and improves over the next 6 months [29]. However, our study sample is more representative of the population needing care than the previous study, as it is selected from men and women who registered at 11 sites after diagnosis through VCT, antenatal testing or as a result of illness. The study cited was of 160 women enrolled into a cohort while HIV-uninfected, who were diagnosed in the acute infection stage.

The trauma of an HIV diagnosis can affect social functioning and mental health in addition to the implications for physical health, which may be as a result of the underlying infection or the psychosomatic impact of knowledge of HIV status. In a study in South Africa, 55% of people with HIV had depressive symptoms just before their diagnosis [30]. Patients with depressive symptoms are less likely to have a CD4 test or to return for the result, which prevents or delays their entry into care [30]. We have demonstrated that while mental-health related quality of life improves in the three months following diagnosis, interpersonal problems remain a great burden. This reflects the highly stigmatised nature of living with HIV. ‘Interpersonal problems’ in the POS comprise two questions: ‘have you been able to share how you are feeling’ and ‘have you had enough help and advice for your family to cope’. Non-disclosure of HIV status may have limited participants’ opportunities to share feelings or provide their family with the right help. The need for emotional support has been shown to be a key motivator for disclosure to family members [31].

Poorer physical function was predictive of a worse response in physical health score and interpersonal problems. Given that late presentation with advanced disease is still relatively common [4], those with poorer function at diagnosis will require greater attention to achieve benefit from entering care and improvements in self-report wellbeing, and should be considered at increased risk. The finding may also reflect added barriers for people with disabilities [32].

In low- and middle-income countries, depression and other mental, neurological and substance use disorders are common among people living with HIV but diagnosis and treatment remains poor [33]. HIV service providers lack confidence and understanding of mental health care [34, 35]. There is growing evidence that rather than being a task for specialist referral, lay health workers can be trained to manage common mental disorders in a primary care setting [36, 37], including care for people with HIV [38].

There are a number of limitations to our study. The cohort length of three months is relatively short, but it covers the crucial period immediately after diagnosis when decisions about care must be made and habits of adherence established [9]. CD4 count was missing from half the sample, almost entirely in Uganda, which, indicates non-random programmatic variation [28]. CD4 was not included in the multilevel models as it would have skewed the sample as well as reducing the sample size, which was already relatively small. Many variables that could affect multidimensional problems or quality of life were not collected and so could not be adjusted for. We note that poorer health-related quality of life and poorer mental health are associated with attrition from care [39], and therefore there is a risk of attrition bias in our sample which would cause outcomes to appear to improve over time. While the use of patient-reported outcomes is a strength of this study, patient-reported clinical details may be a limitation. ART data is patient-reported and clinically assessed ART eligibility was not recorded. We used consecutive sampling, which is non-probabilistic and can be biased, although it is more representative than most other convenience methods.

## Conclusions

Our study has revealed that while improvements in patient-reported outcomes are experienced in the three months following HIV diagnosis, a high burden persists, particularly for psychosocial problems. The benefits of entering care are associated with socio-demographic characteristics such as country, age and poverty. The WHO definition of health, established in 1948, remains relevant: physical, psychological and social wellbeing [40]. Low-cost approaches to person-centred care should be considered for people on ART, especially given the highest burden of interpersonal problems [41]. While access to testing, treatment and care is expanding under current policy, it is important that the psychosocial aspect of HIV disease is not ignored [42].

## Abbreviations

APCA: African Palliative Care Association
KEMRI: Kenya Medical Research Institute
PEPFAR: President’s Emergency Plan for AIDS Relief
UNCST: Uganda National Council for Science and Technology
USG: United States Government
WHO: World Health Organisation
